# Comparison of posterior support strategies with pterygoid implants for full-arch implant rehabilitation in the atrophic maxilla: a finite element study

**DOI:** 10.1186/s12903-025-07060-5

**Published:** 2025-10-31

**Authors:** Ummugulsum Coskun, Nuray Yilmaz Altintas

**Affiliations:** Department of Oral and Maxillofacial Surgery, Altinbas University Faculty of Dentistry, Istanbul, 34147 Turkey

**Keywords:** Dental implants, Finite element analysis, Alveolar bone loss, Stress, Mechanical, Maxilla

## Abstract

**Background:**

Rehabilitation of the atrophic maxilla is challenging due to bone resorption and anatomical limitations. Although the All-on-Four concept offers a predictable treatment strategy, posterior cantilevers may increase biomechanical risk. Pterygoid implants have been proposed as an alternative to enhance posterior support without the need for bone grafting. This study aimed to compare stress distributions in bone and prosthetic components using three different implant-supported treatment strategies for the atrophic maxilla.

**Methods:**

Three three-dimensional finite element models were developed based on cone-beam computed tomography scans of an edentulous maxilla. Model 1 included four implants following the All-on-Four protocol. Model 2 consisted of five implants, including two pterygoid implants for posterior support. Model 3 comprised six implants, also including two pterygoid implants. Stress distributions in cortical and trabecular bone, implants, abutments, and prosthetic frameworks were analyzed under oblique occlusal loading conditions.

**Results:**

Model 3 demonstrated the most favorable biomechanical profile, with reduced maximum and minimum principal stresses and strain values in both cortical and trabecular bone. Model 1 exhibited the highest stress concentrations, particularly around posterior implants (up to 110 MPa) and prosthetic components (28–45 MPa), likely due to the cantilever effect. In contrast, Model 3 showed lower maximum principal stress in the cortical bone (0.4 MPa) compared to Model 1 (1.54 MPa). Additionally, the von Mises stress in the first and second implants decreased in Model 3 (28 MPa and 63.6 MPa, respectively) compared to Model 1 (39 MPa and 110.5 MPa) and Model 2 (20 MPa and 80.0 MPa).

In terms of strain distribution, all models remained within physiological thresholds but Model 3 exhibited a more balanced and uniform strain pattern, particularly around posterior implant sites. This suggests improved load transfer and reduced risk of biomechanical overload.

**Conclusions:**

Increasing the number of implants and incorporating pterygoid implants enhances biomechanical stability in the atrophic maxilla. These strategies reduce stress and strain concentrations in both bone and prosthetic components and offer a less invasive alternative to bone grafting procedures. Optimizing implant number and posterior support is critical for improving long-term success in full-arch implant rehabilitation.

## Introduction

Dental implants are widely recognized as a successful treatment option for the replacement of missing teeth [1, 2]. In cases where sufficient bone is available in the maxilla, the success rate of implant rehabilitation has been reported to range between 84% and 92%. However, the presence of atrophic bone in the maxillary region is a common clinical condition that poses challenges for conventional implant placement. Alveolar ridge resorption, pneumatization of the maxillary sinuses, anatomical structures such as the nasal fossae and the nasopalatine canal, and poor bone quality are among the main factors that complicate implant placement in the maxilla [3]. To overcome these limitations, various treatment strategies have been developed for the rehabilitation of the atrophic maxilla. These include bone graft augmentation, guided bone regeneration, alveolar distraction osteogenesis, sinus floor elevation, the placement of implants in alternative anatomical sites, the use of tilted implants, and short implants [4]. However, grafting procedures have certain drawbacks, such as the need for multiple surgical interventions, increased patient morbidity, a higher risk of complications, longer treatment duration, increased costs, and lower patient acceptance [5, 6].

The “All-on-Four” technique was introduced by Malo et al. to facilitate the placement of fixed prostheses in patients with the atrophic maxilla. This concept involves a full-arch fixed prosthesis supported by four implants, with two placed in the anterior region and two tilted implants in the posterior region [7]. Tilting the posterior implants distally allows for more posterior implant positioning and enables the use of the cortical bone of the sinus and nasal fossa walls to enhance implant anchorage [7, 8]. This configuration increases the inter-implant distance, shortens the cantilever length, and results in a more favorable stress distribution [8].

Pterygoid implants were first described by Tulasne and Tessier in 1989 as a means to take advantage of the existing bone structure in the pterygomaxillary region. According to Tulasne, posterior atrophic maxilla preserves approximately 80% of the original bone corridor, which is considered sufficient for placing an implant 13–20 mm in length [9]. Ideally, a pterygoid implant should extend into the dense cortical pterygoid plate of the sphenoid bone, pass through the maxillary tuberosity, and engage the pterygomaxillary junction, where it merges with the pyramidal process of the palatine bone [10].

Compared to other techniques, pterygoid implants offer several advantages: they provide reliable anchorage in the posterior atrophic maxilla without the need for sinus lifting or bone grafting, thereby improving stability and long-term success rates. Additionally, they eliminate the need for a posterior cantilever and enhance axial loading [11, 12]. The anteroposterior angulation of pterygoid implants generally ranges from 45° to 70° relative to the Frankfort horizontal plane, while the buccopalatal angulation ranges between 10° and 15°. Anatomical and radiological studies have recommended a minimum implant length of 13 mm to ensure anchorage in the dense cortical pterygoid plate. However, other studies have suggested that longer implants (13–20 mm) may be associated with higher success rates in this region [13, 14].

The finite element (FE) method is a numerical approach widely used to analyze and simulate physical phenomena, helping to reduce the need for physical prototypes and enabling the development of more efficient and cost-effective solutions through component optimization. In dentistry, finite element analysis (FEA) has been extensively applied to investigate the mechanical behavior of implants under functional loading and to assess stress and strain distribution in implants and surrounding bone [15–17]. Mechanical loading induces adaptive changes in bone, which influence stress and strain patterns at the bone–implant interface and impact osseointegration and remodeling. Numerical modeling of these effects improves understanding and supports the development of strategies to enhance the long-term success of implant treatments [18, 19].

Recent years have seen a growing focus on the biomechanical and clinical benefits of incorporating pterygoid implants for posterior support in full-arch rehabilitations, particularly in the atrophic maxilla [20–22]. These investigations highlight the critical role of posterior anchorage in minimizing cantilever effects, improving load distribution, and enhancing the long-term success of implant-supported prostheses. Several recent studies have reported improved clinical outcomes and mechanical stability when using pterygoid implants in challenging maxillary conditions [23, 24]. Although the biomechanical benefits of pterygoid implants have been addressed in previous research, there remains a lack of comprehensive comparative analyses assessing both stress and strain distributions across different posterior support configurations incorporating pterygoid implants. Previous FEAs have typically focused on conventional All-on-Four models or individual posterior support strategies. To the best of our knowledge, no prior study has comprehensively compared the stress and strain distribution patterns among configurations such as the conventional All-on-Four and the All-on-Five and All-on-Six models that incorporate pterygoid implants. This study introduces a novel comparison framework by evaluating stress and strain distributions across three posterior implant configurations with pterygoid support. This dual-assessment approach provides a more comprehensive and physiologically relevant insight into the mechanical behavior of full-arch implant rehabilitations in the atrophic maxilla. Therefore, the aim of this study was to evaluate and compare the stress distributions in dental implants, cortical and trabecular bone, abutments, and prosthetic components and the strain distribution specifically in cortical and trabecular bone under occlusal loading using FEA, based on three distinct treatment models in the atrophic maxilla: the All-on-Four protocol and the All-on-Five and All-on-Six models using pterygoid implants.

## Materials and methods

This study was supported by the Scientific Research Projects Coordination Unit of Altınbas University (PB2023-DIS-3). All analyses were carried out in collaboration between Altınbas University and Ay Tasarım Ltd. Using the FEA method, stress distribution in cortical and trabecular bone, implants, and prosthetic components was evaluated under occlusal loading based on three different dental implant placement models in a completely edentulous and atrophic maxilla. A static linear analysis was performed and a three-dimensional FEA model was used as the basis for the study. This is not a clinical trial; therefore, trial registration was not applicable.

### Model configurations

Model 1: A simulation based on the All-on-Four concept was developed. The anterior implants were placed axially in the lateral incisor-canine regions. The posterior implants were bilaterally placed anterior to the maxillary sinus wall and tilted distally at an angle of 30° relative to the occlusal plane.

Model 2: This configuration included five implants. One axial implant was positioned in the left lateral incisor-canine region. Two tilted implants were placed in the premolar region just anterior to the maxillary sinus, each angled distally at 30°. Additionally, two pterygoid implants were placed in the regions corresponding to teeth 17 and 27, angulated anteriorly at 70°.

**Fig. 1:**
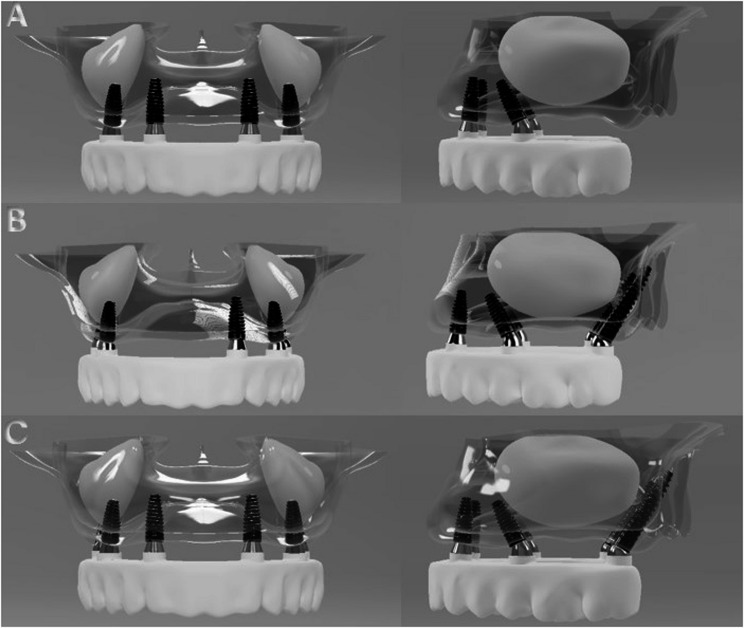
Models showing three different dental implant placement strategies analyzed in the study: (**A**) Model 1 (All-on-Four); (**B**) Model 2 (All-on-Five with pterygoid implants); (**C**) Model 3 (All-on-Six with pterygoid implants)

Model 3: This model involved six implants. Two axial implants were placed in the lateral incisor-canine regions. Two tilted implants were positioned in the premolar region, just anterior to the maxillary sinus, each angled distally at 30°. Additionally, two pterygoid implants were placed at the 17 and 27 tooth regions, each angulated anteriorly at 70°. The configurations of all three models are illustrated in Fig. 1.

### Geometry acquisition and modeling

**Fig. 2:**
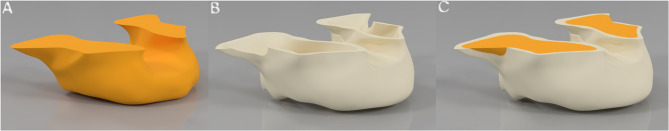
The bone modeling process: (**a**) trabecular bone model; (**b**) cortical bone model; (**c**) completed maxilla model

The maxillary jaw geometry used in this study was based on a pre-existing 3D model previously constructed from anonymized, retrospective CBCT data of a fully edentulous maxilla. The original model was generated from 601 axial slices acquired using the ILUMA CBCT system (3 M Imtec, USA), with a slice thickness of 0.2 mm. The DICOM 3.0 data were imported into 3D-Doctor software (Able Software Corp., USA) for segmentation and preprocessing. Bone tissue was segmented interactively, and a 3D model was created using the “3D Complex Render” method. To replicate the trabecular architecture, an offset technique was used to simulate internal bone morphology and ensure realistic force transmission. The stages of model generation are shown in Fig. 2. To ensure anatomical fidelity, CBCT-derived models were edited in Blender (Blender Foundation, Netherlands) for refinement. A solid maxillary model was created and exported for FEA. Linear tetrahedral elements were used for meshing to balance computational efficiency with anatomical complexity. The solid model was built and assembled using Fusion 360 (Autodesk Inc., USA), where all components were integrated to reflect biological morphology.

### Implant and prosthetic component modeling

All implants, abutments, and screw-retained components were modeled individually in SolidWorks (Dassault Systèmes, France) based on precise manufacturer specifications. Straumann Bone Level Tapered (BLT) implants (Institut Straumann AG, Basel, Switzerland) were used in both the anterior and premolar regions. Implants in the anterior region measured 10 mm in length and 4.1 mm in diameter; premolar region implants measured 14 mm in length and 4.1 mm in diameter. In all groups, premolar implants were positioned at a 30° distal angulation, with apices aligned with teeth 14 and 24, and necks aligned with teeth 15 and 25. Pterygoid implants used were JDentalCare implants (JDentalCare S.r.l., Modena, Italy), 4.0 mm in diameter and 20 mm in length. These implants originated from the 17 and 27 tooth regions, passed through the pyramidal process of the palatine bone, and ended at the pterygoid process of the sphenoid bone. Entry points were defined in these regions, and bone quality was classified as D3. Implants were angulated at 70° anteroposteriorly and 15° buccopalatally relative to the Frankfort horizontal plane [12–14]. Apices reached high-density cortical regions of the pyramidal process and pterygoid bone. A 1 mm thick cortical bone layer was modeled around the pterygoid implants, and bicortical stabilization was achieved with 1 mm penetration into the pterygoid plate.

Prosthetic frameworks were modeled using zirconia, based on the Toronto protocol. Lithium disilicate ceramic crowns (IPS e.max, Ivoclar) were individually modeled and connected at contact points. After solid model construction, all components were assembled in SolidWorks to reflect biological morphology. Cortical and trabecular bone, implants, abutments, substructures, and restorations were integrated to preserve anatomical accuracy and prosthetic continuity.

### Material properties

All materials were modeled as linear, homogeneous, and isotropic. The elastic modulus and Poisson’s ratio of each material were defined based on validated literature sources as summarized in Table 1 [25, 26]. These assumptions allowed for consistent mechanical behavior across all structures within the simulation.

**Table 1:** Element and node numbers according to models

	Model 1	Model 2	Model 3
Nodes	126,421	213,092	329,231
Elements	581,373	1,006,605	1,637,542

### Mesh generation and convergence analysis

**Fig. 3:**
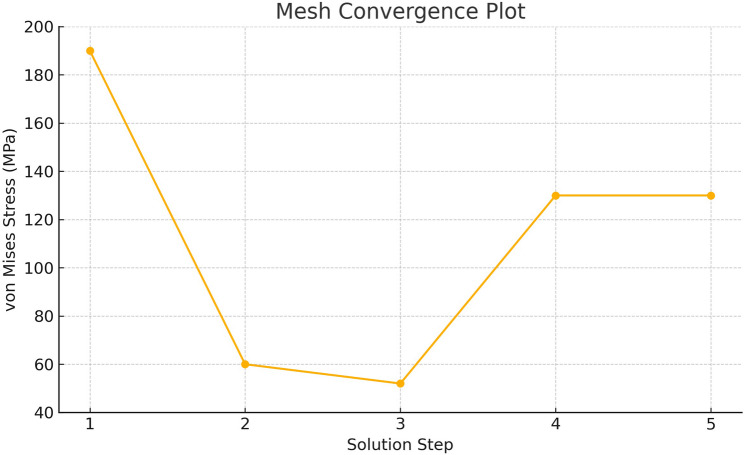
The graph illustrates von Mises stress values (MPa) obtained from five different mesh densities to validate mesh independence. A convergence threshold of 10% was applied, and each solution step represents a progressively refined mesh. The stress values stabilized between Step 4 and Step 5, confirming that the final mesh configuration was adequate for further analysis

Mesh generation and optimization were performed using Fusion 360. The average element size was set to 10% of the model dimension, with a minimum element size at 10% of the average. The mesh employed linear tetrahedral elements, which are standard in dental biomechanics studies due to their balance of efficiency and precision. A mesh convergence analysis was performed using adaptive mesh refinement (AMR), with von Mises stress values used as the convergence criterion. A maximum of four refinement steps was allowed, with 25% of elements adaptively refined at each step. Convergence tolerance was set to 10%. The refinement process yielded increased mesh density in high-stress regions. The convergence plot, presented in Fig. 3, confirmed result stability across iterations. To ensure mesh independence, a convergence analysis was performed by solving the model using five progressively refined mesh densities. Figure 3 shows the corresponding von Mises stress values, demonstrating that the results stabilized between Step 4 and Step 5, confirming that mesh independence was achieved and that the final mesh configuration was suitable for further analysis. No curved elements were used. Additional mesh control parameters included a maximum adjacent mesh size ratio of 2.8, a maximum aspect ratio of 18, and a maximum turn angle of 60°. Mesh size scaling was applied part-specifically to preserve geometric detail. The final number of elements and nodes presented in Table 2 reflects the mesh configuration selected after the convergence assessment. This ensures an optimal balance between computational efficiency and numerical accuracy. As expected, the number of elements exceeded the number of nodes due to element-node sharing, which is typical in FEA.

**Table 2:** Elastic modulus and poisson’s ratio values of the materials

Materials	Young’s Modulus (MPa)	Poisson’s Ratio (*n*)
Cortical bone	13,700	0.30
Trabecular bone	1370	0.30
Titanium	110,000	0.35
Zirconia	205,000	0.22
Ceramic	95000.00	0.20

### Boundary and loading conditions

**Fig. 4:**
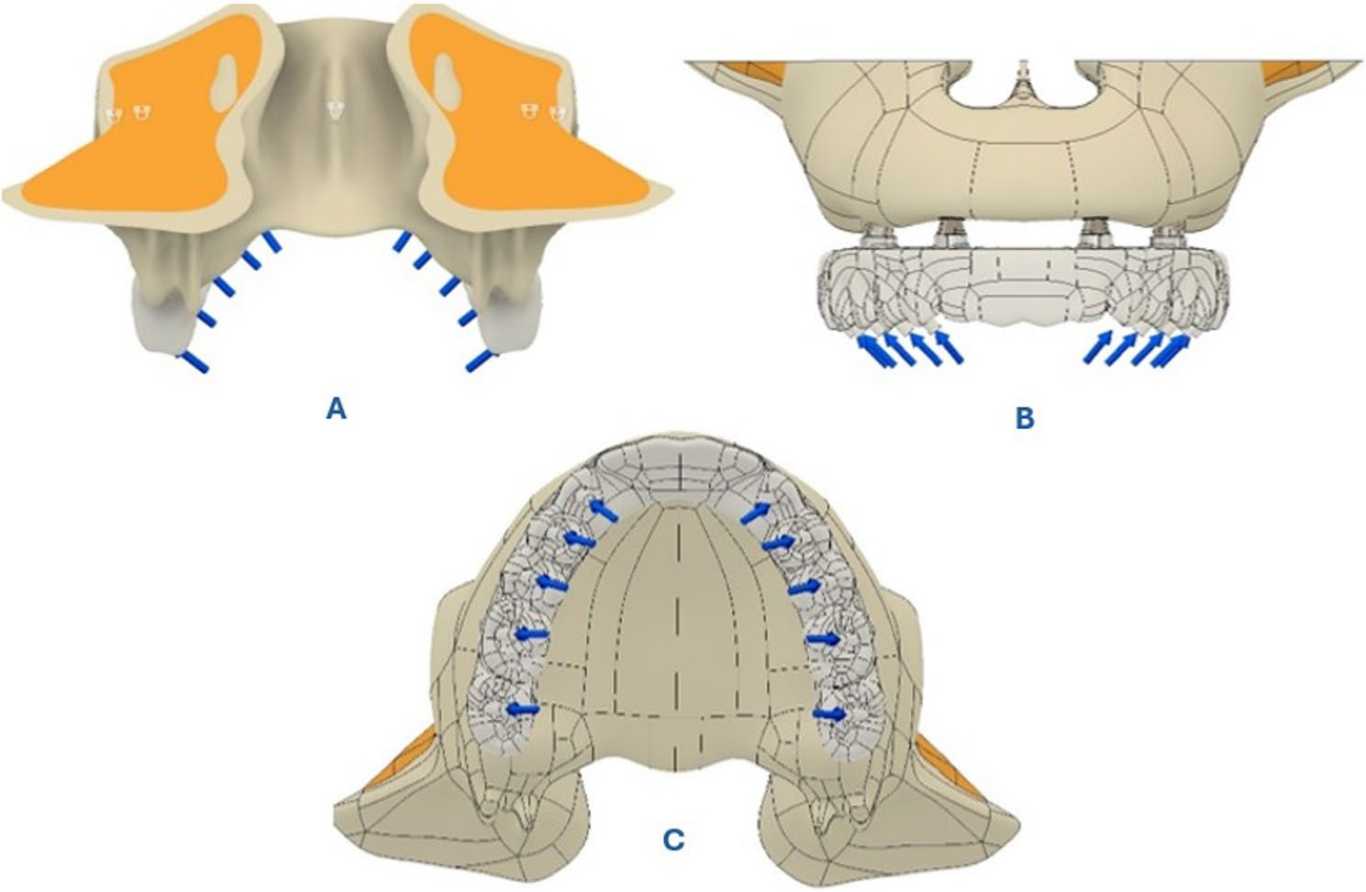
Occlusal load application and boundary conditions in the finite element model. **A** Posteroinferior view showing the fixation of the maxilla. **B** Frontal view illustrating bilateral occlusal loads applied at a 45° palatobuccal angle. **C** Occlusal view indicating load application sites. Forces of 50 N, 100 N, 100 N, 150 N, and 150 N were respectively applied to the regions corresponding to teeth numbers 3, 4, 5, 6, and 7

Boundary conditions were applied by fully constraining the superior-posterior region of the maxilla to simulate physiological fixation. During the loading condition setup, the model edges were restricted along the x, y, and z axes to eliminate displacement in any direction. All structures were assumed to be perfectly bonded to ensure optimal load transmission [27, 28]. Occlusal forces were applied bilaterally at a 45° angle in the palatobuccal direction. Specifically, forces of 50 N, 100 N, 100 N, 150 N, and 150 N were applied to the regions corresponding to teeth numbers 3, 4, 5, 6, and 7, respectively [26, 29]. These modeling strategies ensured a physiologically accurate simulation of full-arch biomechanics. The loading configuration is illustrated in Fig. 4.

### Analysis

Stress in implants, abutments, frameworks, and superstructures was analyzed using von Mises stress (σ_VM_) values. Stresses in trabecular and cortical bone were assessed via maximum principal stress (σ_max_) and minimum principal stress (σ_min_). In the analyses, the highest stress values observed in the structures were taken into account, and all data were recorded in megapascals (MPa). To complement the stress analysis and provide a more comprehensive biomechanical evaluation, maximum principal strain values (expressed in microstrain, µε) were also analyzed for both cortical and trabecular bone. The strain values were extracted from specific regions surrounding the implants on both the right and left sides for all three models. Stress and strain distributions were automatically calculated by the software and visualized through color-coded magnitude maps.

## Results

As FEA yields deterministic outcomes based on defined geometry, boundary conditions, and material properties, statistical comparisons were not applicable in this study. This approach is consistent with prior FEA-based biomechanical investigations [26, 30, 31].

### Stress values in bone tissues

A color scale-based representation was used to evaluate the distribution of σ_max_ in the cortical and cancellous bone in more detail. In this scale, stress values increased as the color shifted from blue to red, with red areas indicating higher stress concentrations. Similarly, a color-coded scale was used to assess the distribution of σ_min_. In this case, increasing stress values were represented by a shift in color from red to blue, where darker shades of blue indicated higher minimum stress levels. Additionally, the obtained values were graphically expressed using heatmaps.

**Fig. 5:**
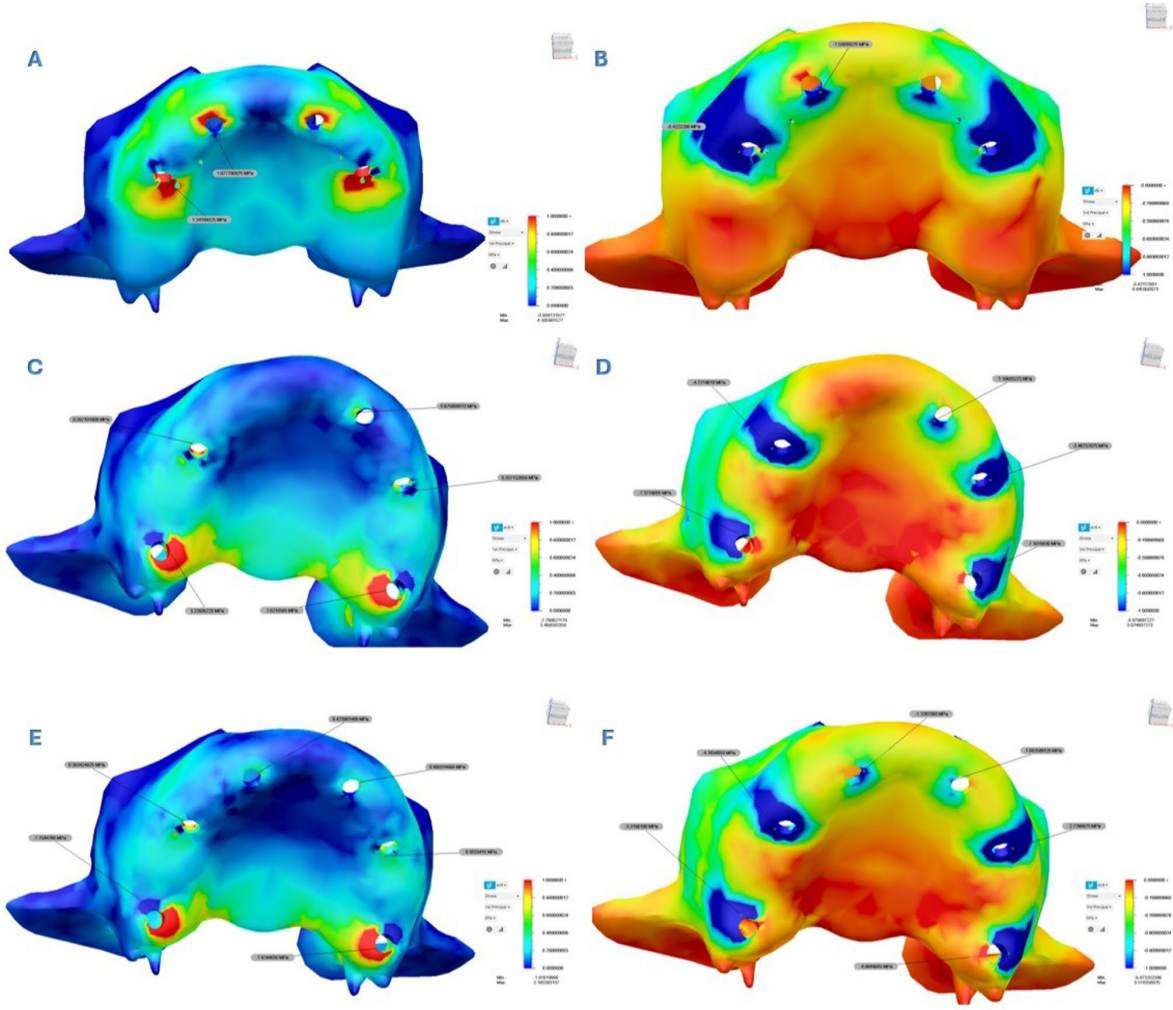
Principal stress distributions in the cortical bone Maximum principal stresses (**A**, **C**, **E**) and minimum principal stresses (**B**, **D**, **F**) are shown for Models 1, 2, and 3, respectively

**Fig. 6:**
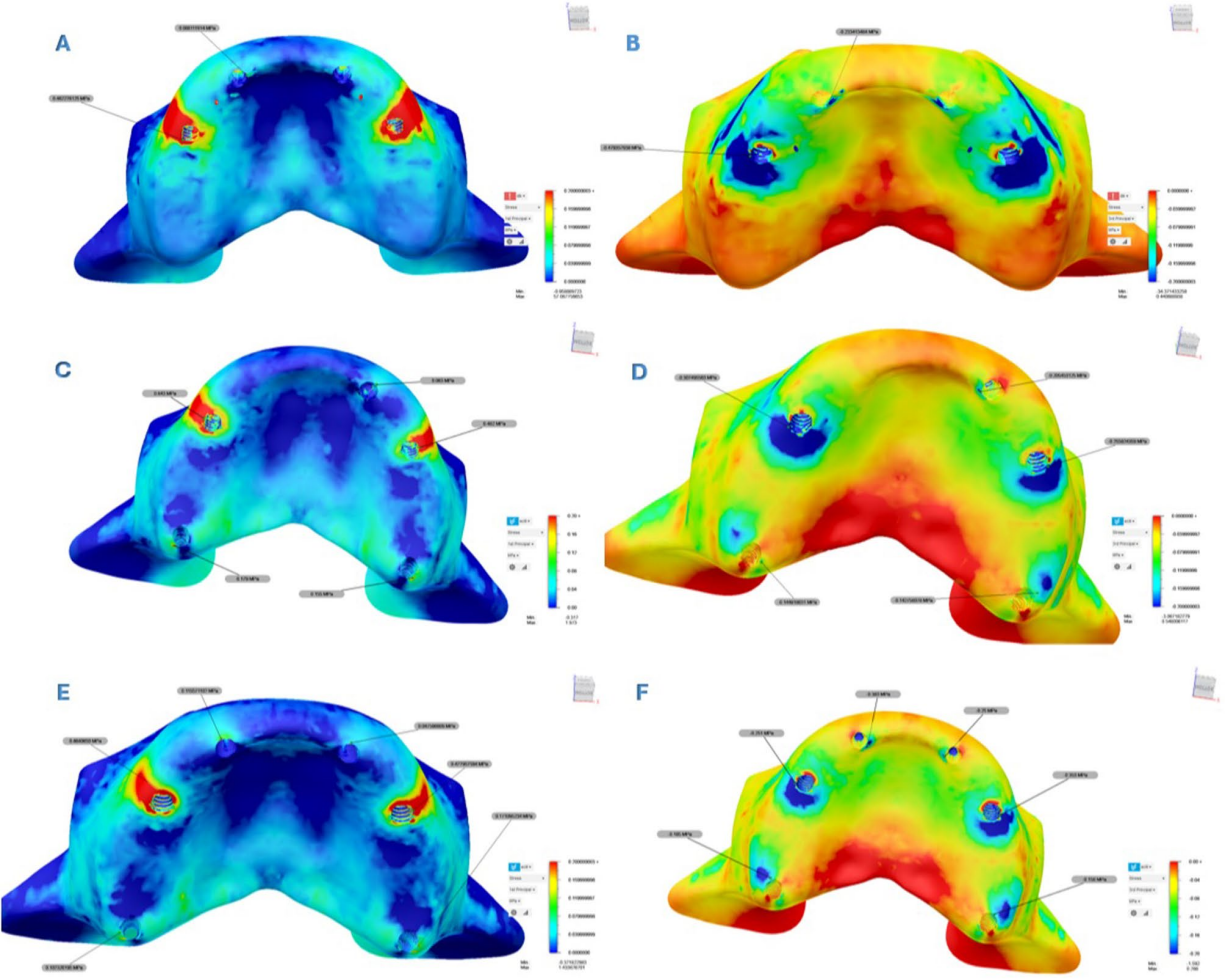
Principal stress distributions in the trabecular bone Maximum principal stresses (**A**, **C**, **E**) and minimum principal stresses (**B**, **D**, **F**) are shown for Models 1, 2, and 3, respectively

**Fig. 7:**
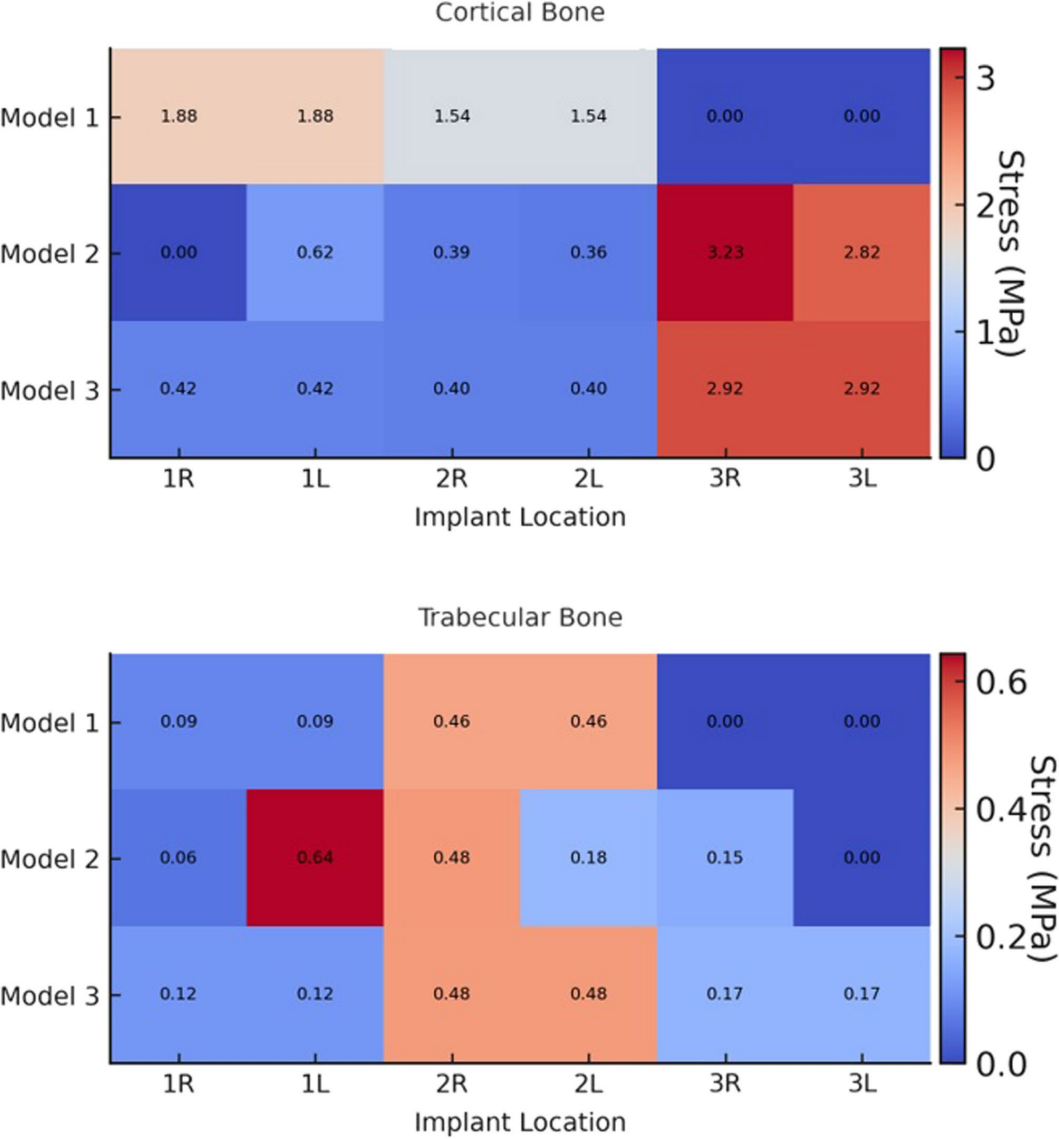
Heatmaps showing the distribution of maximum principal stress (MPa) in cortical and trabecular bone for different implant locations, anterior right and left (1R, 1 L), premolar right and left (2R, 2 L), and pterygoid right and left (3R, 3 L) regions, across Models 1, 2, and 3

**Fig. 8:**
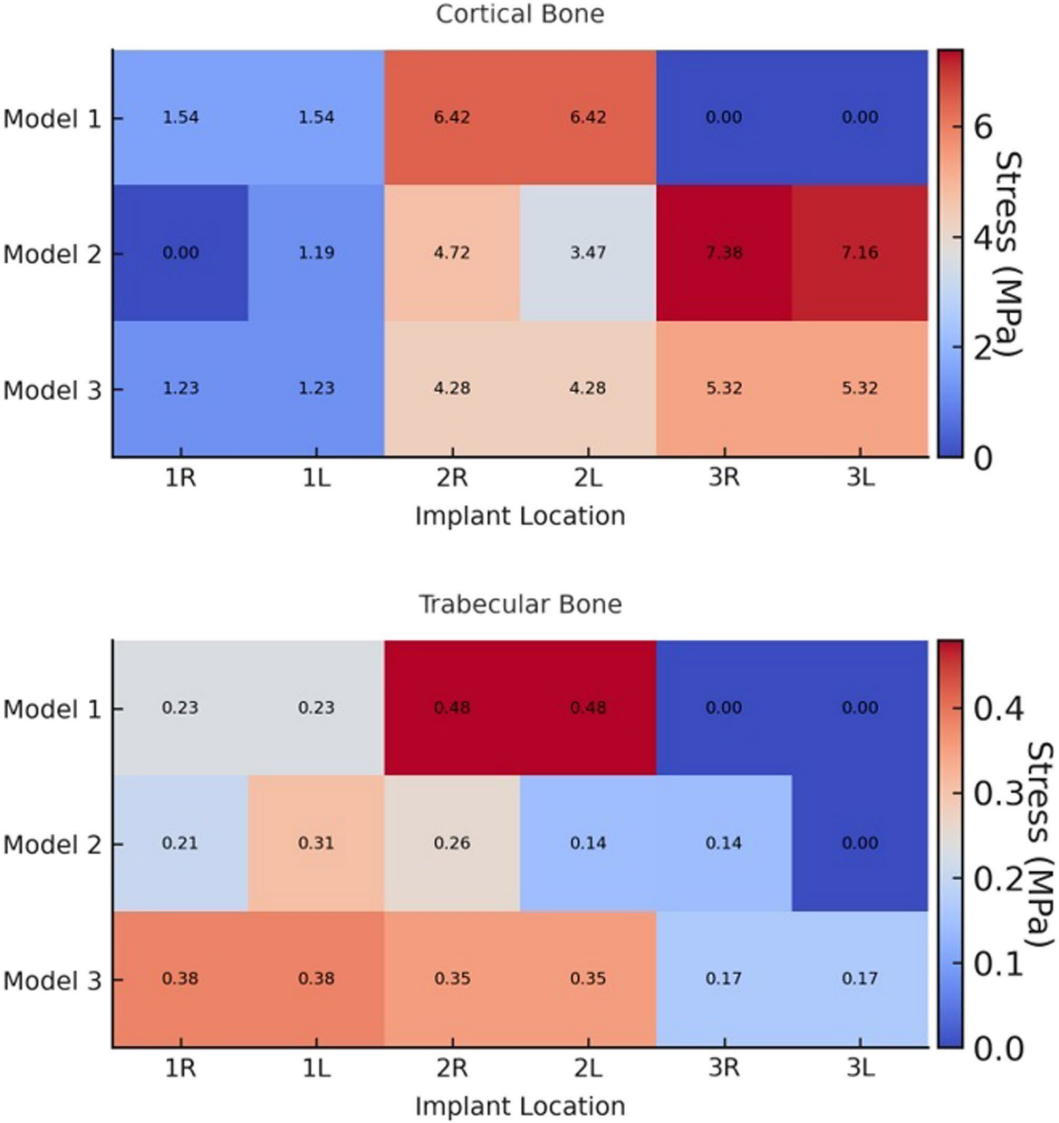
Heatmaps showing the distribution of minimum principal stress (MPa) in cortical and trabecular bone for different implant locations: anterior right and left (1R, 1 L), premolar right and left (2R, 2 L), and pterygoid right and left (3R, 3 L) regions, across Models 1, 2, and 3

Analysis revealed that both σ_max_ and σ_min_ values were consistently higher in cortical bone compared to trabecular bone. Stress distribution was generally concentrated around the implant sockets in the posterior region of the cortical bone. The detailed σ_max_ and σ_min_ values for each design are presented in Tables 3 and 4, and their spatial distribution is illustrated in Figs. 5 and 6. In addition to these numerical evaluations, the stress distributions were also visualized using heatmaps to enhance graphical interpretation. These visual representations are shown in Figs. 7 and 8.

**Table 3:** σ_max_ values in trabecular and cortical bone for each model (MPa)

Implant Location		1 st implant		2nd implant		3rd implant	
		Right	Left	Right	Left	Right	Left
**Model 1**	Cortical Trabecular	1.87779 0.08811	1.87779 0.08811	1.54189 0.46227	1.54189 0.46227		
**Model 2**	Cortical Trabecular		0.62500 0.063	0.38710 0.643	0.357132 0.482	3.22605 0.179	2.82105 0.155
**Model 3**	Cortical Trabecular	0.42398 0.11557	0.42398 0.11557	0.40031 0.47795	0.40031 0.47795	2.92446 0.17109	2.92446 0.17109

*σ*_*max*_: *Maximum principal stress*

**Table 4:** σ_min_ values in trabecular and cortical bone for each model (MPa)

Implant Location		1 st implant		2nd implant		3rd implant	
		Right	Left	Right	Left	Right	Left
**Model 1**	Cortical Trabecular	1.53859 0.23341	1.53859 0.23341	6.42222 0.47935	6.42222 0.47935		
**Model 2**	Cortical Trabecular		1.18603 0.205453	4.72146 0.30749	3.46732 0.255824	7.37780 0.14481	7.16108 0.142758
**Model 3**	Cortical Trabecular	1.22623 0.383	1.22623 0.383	4.28340 0.353	4.28340 0.353	5.31601 0.165	5.31601 0.165

*σ*_*min*_: *Minimum principal stress*

Comparative analysis among Models 1, 2, and 3 showed that Models 2 and 3 exhibited similar stress distribution patterns, while Model 1 demonstrated the highest stress values for both σ_min_ and σ_max_. Notably, in Model 1, σ_max_ values around the posterior implants were significantly higher than in the other models. When comparing Models 2 and 3, it was observed that the σ_min_ and σ_max_ values were similar in all implants on the left side of the maxilla. However, on the right side, Model 2 demonstrated higher stress values compared to Model 3.

In all models, σ_max_ in cortical bone were concentrated around the posterior implants, especially on the distopalatinal surface of the alveolar sockets. In the premolar region, stresses were most prominent on the buccal and distal surfaces. In anterior implants, stress accumulation was primarily observed on the buccal surfaces. In trabecular bone, the highest σ_min_ and σ_max_ levels were typically observed on the buccal surfaces of the implant sockets in the premolar region, while in the anterior implants, they were more pronounced on the palatal side. In Model 2, which features an asymmetric distribution, cortical bone stress was higher on the right side, where fewer implants were placed.

**Fig. 9:**
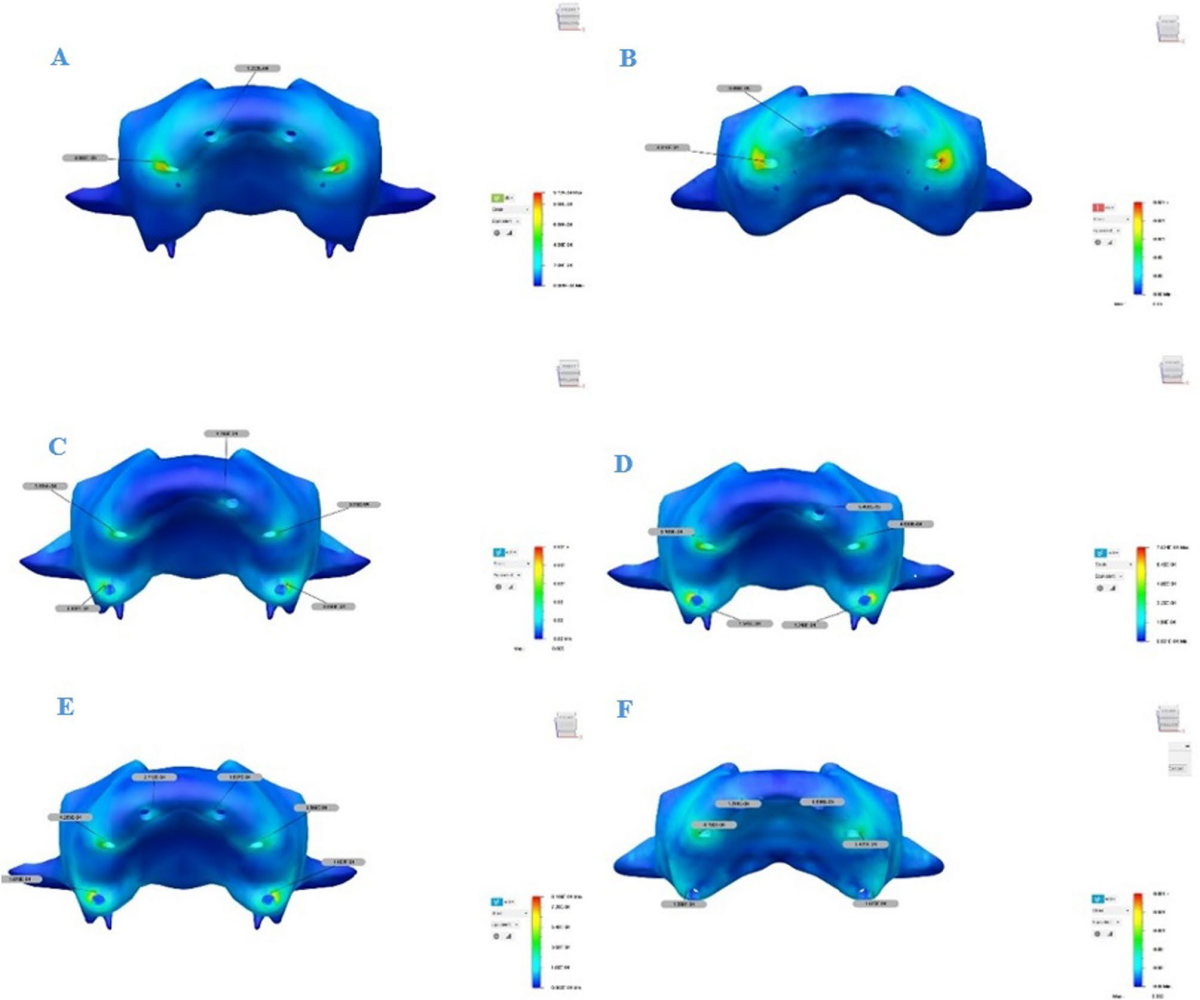
Maximum principal strain distributions in cortical and trabecular bone across all models. (**A**, **C**, **E**) show the cortical bone strain maps for Models 1, 2, and 3, respectively. (**B**, **D**, **F**) show the corresponding trabecular bone strain maps for the same models. Color-coded heatmaps represent microstrain (µε) values, ranging from low (blue) to high (red) levels.

**Fig. 10:**
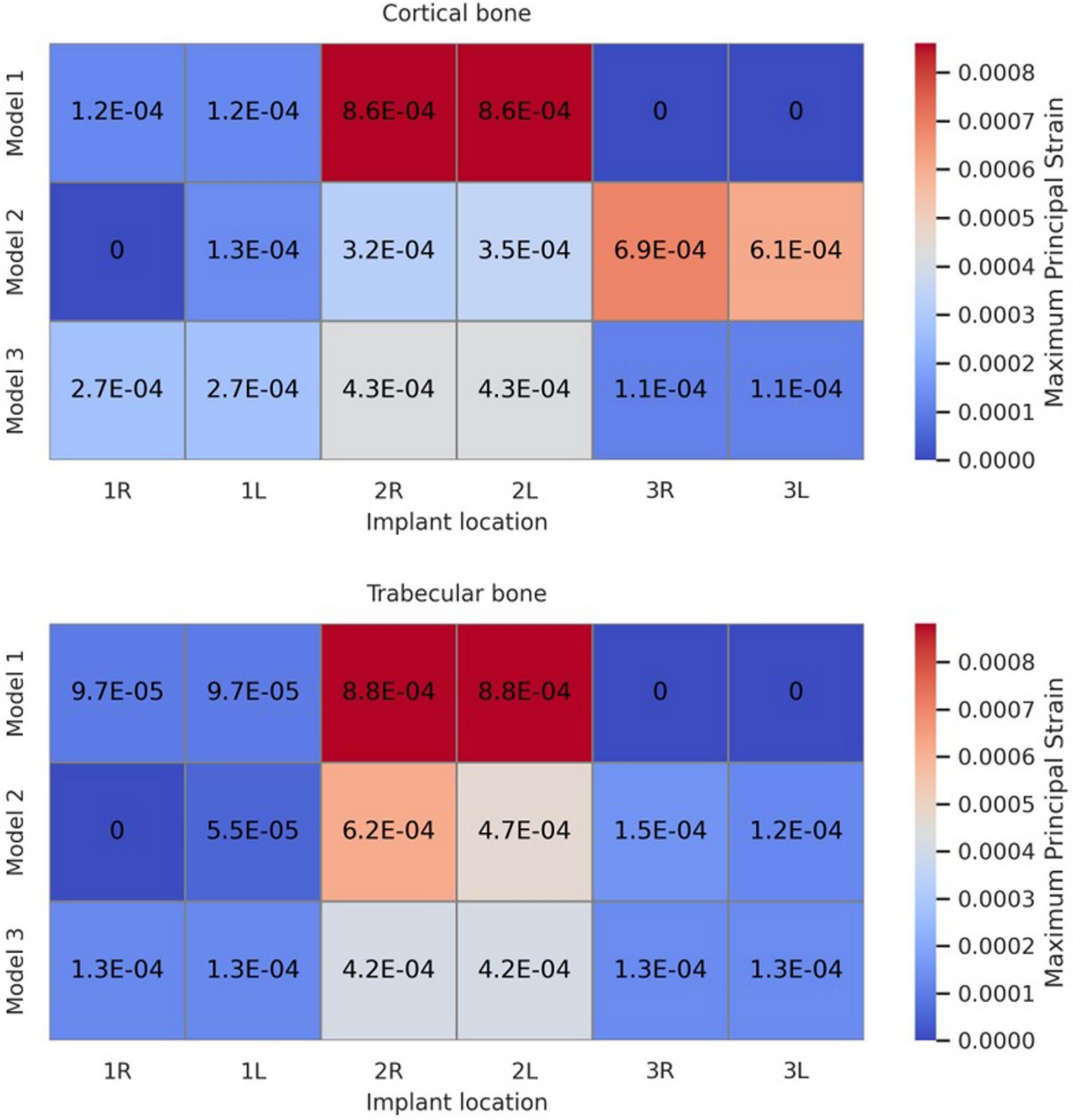
Heatmaps showing the distribution of maximum principal strain in cortical and trabecular bone for different implant locations: anterior right and left (1R, 1 L), premolar right and left (2R, 2 L), and pterygoid right and left (3R, 3 L), across Models 1, 2, and 3 Strain values are given in scientific notation. Red areas indicate higher strain concentrations, while blue areas represent lower strain levels

Maximum principal strain values were evaluated in both cortical and trabecular bone surrounding the implants in all three models. In all models, the highest maximum principal strain values were observed in cortical and trabecular bone around the distal implants of Model 1, reaching up to 8.602E–04 (860 µε). Strain was primarily concentrated in the buccal bone surrounding the distal implants. In Model 2, strain values in cortical bone increased progressively toward the posterior region, whereas tensile strain in trabecular bone was more prominent in the anterior region. Strain was mainly concentrated in the buccal bone near the premolar and pterygoid implants, whereas it was more prominent in the palatal bone around the anterior implants. Model 3 exhibited a more balanced strain distribution overall. However, the highest strain values in both cortical and trabecular bone were detected in the second premolar regions. Similar to Model 2, strain concentrated in the buccal bone near the premolar and pterygoid implants, while in the anterior region, it was predominantly located in the mesiopalatal bone. The strain distributions observed across all maxillary models are illustrated in Fig. 9. All observed strain values remained within the physiological limits. The numerical data are presented in Table 5, and spatially visualized as color-coded heatmaps in Fig. 10. These findings indicate that the use of pterygoid implants in Models 2 and 3 contributed to reduced strain levels in both cortical and trabecular bone when compared to the conventional All-on-Four configuration.

**Table 5:** Maximum principal strain values in cortical and trabecular bone

Implant Location		1 st implant		2nd implant		3rd implant	
		Right	Left	Right	Left	Right	Left
**Model 1**	Cortical Trabecular	1.222E-04 9.698E-05	1.222E-04 9.698E-05	8.602E-04 8.810E-04	8.602E-04 8.810E-04	- -	- -
**Model 2**	Cortical Trabecular	- -	1.293E-04 5.459E-05	3.195E-04 6.169E-04	3.519E-04 4.688E-04	6.887E-04 1.545E-04	6.096E-04 1.248E-04
**Model 3**	Cortical Trabecular	2.712E-04 1.291E-04	2.712E-04 1.291E-04	4.263E-04 4.150E-04	4.263E-04 4.150E-04	1.078E-04 1.308E-04	1.078E-04 1.308E-04

*Values are in scientific notation.*

### Stress values in implant and prosthetic components

**Fig. 11:**
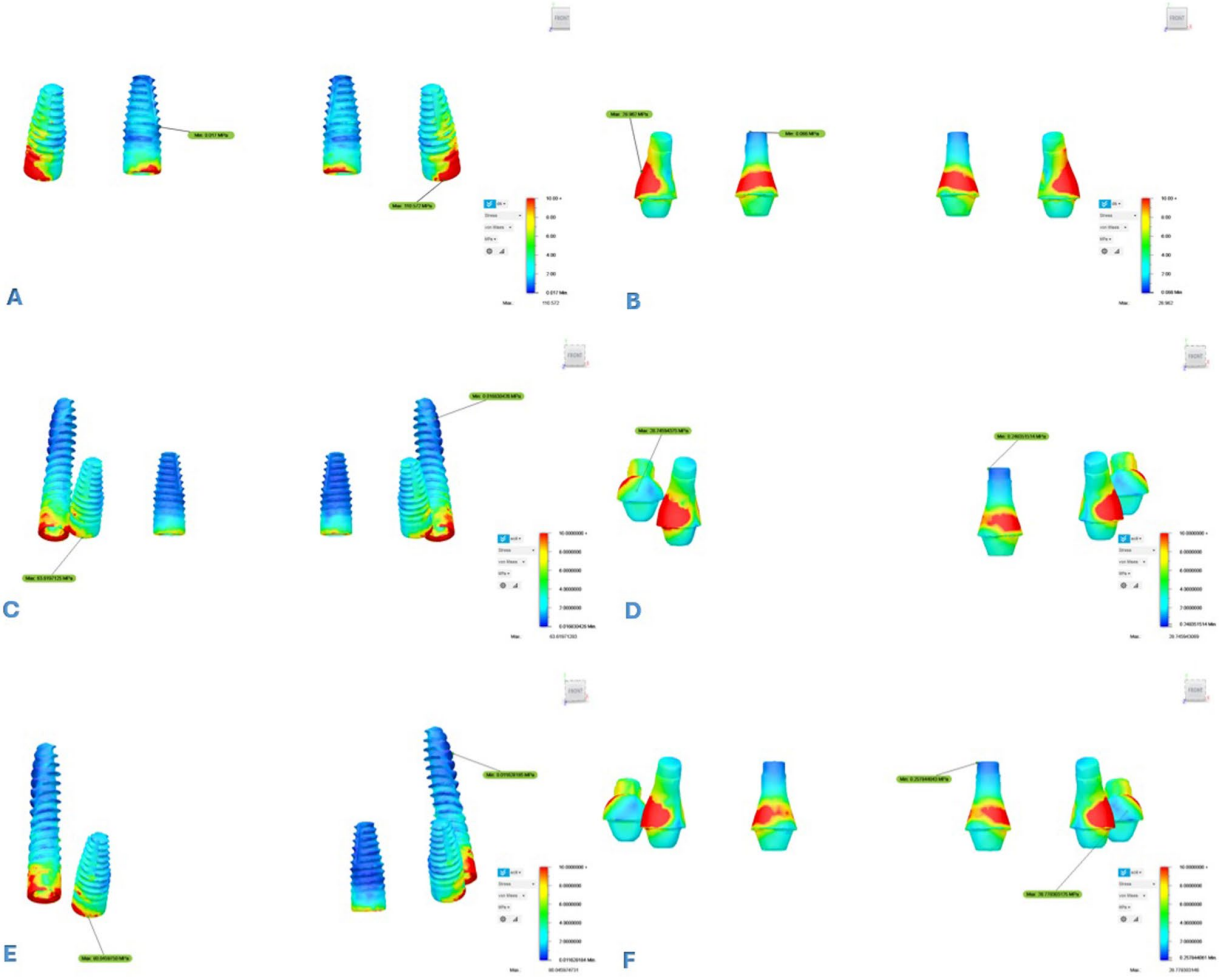
σ_VM_ distributions in implants (**A**, **C**, **E**) and abutments (**B**, **D**, **F**) for Models 1, 2, and 3. Red indicates high stress areas, and blue indicates low stress regions σ_VM_: von Mises stress

**Fig. 12:**
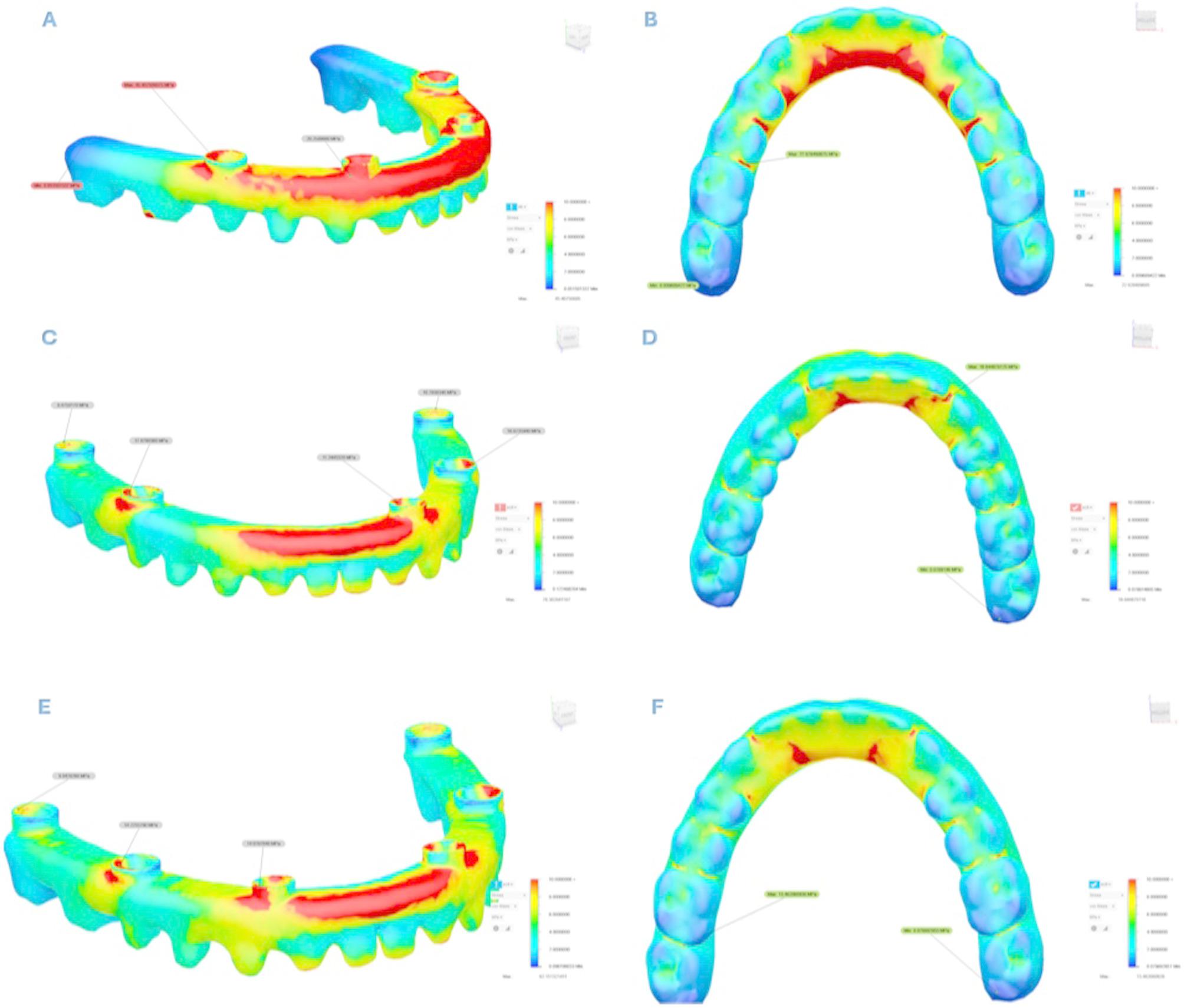
σ_VM_ distributions in prosthetic frameworks (**A**, **C**, **E**) and crowns (**B**, **D**, **F**) for Models 1, 2, and 3. Red areas represent high-stress regions, while blue areas represent low-stress regions σ_VM_: von Mises stress

To assess σ_VM_ distribution in dental implants and prosthetic components, color-coded stress maps were utilized, with stress intensity increasing from blue to red. The corresponding stress values are summarized in Table 6 and visually represented in Figs. 11 and 12.

**Table 6:** σ_VM_ values on implants, abutments and framework for each model (MPa)

Implant Location		1 st implant		2nd implant		3rd implant	
		Right	Left	Right	Left	Right	Left
**Model 1**	Implant Abutment Framework	39.797 17.289 20.25899	39.797 17.289 20.25899	110.572 28.962 45.40750	110.572 28.962 45.40750		
**Model 2**	Implant Abutment Framework		20.9890 13.44097 11.24453	80.04597 27.56566 17.67903	61.73875 24.18778 18.87359	47.54438 28.74594 8.47501	44.43859 27.95766 10.78383
**Model 3**	Implant Abutment Framework	28.31067 13.70538 14.02876	28.31067 13.70538 14.02876	63.61971 25.11984 14.22337	63.61971 25.11984 14.22337	45.82940 28.77830 9.04282	45.82940 28.77830 9.04282

*σ*_*VM*_: *von Mises stress*

In all models, the highest σ_VM_ values were generally observed in the neck regions of the second implants where they contacted the cortical bone. When the anterior implants were examined, the highest surrounding stress was found in Model 1, while the lowest was observed in Model 2. When the right and left implants of Model 2 were compared, the second and third implants on the right side exhibited higher stress values than those on the left side. Additionally, stresses in all implants were found to be concentrated in the neck region and gradually decreased toward the apex.the These distributions are illustrated in Fig. 11.

As the number of implants increased, stress values in both the implants and the prosthetic components decreased. The highest σ_VM_ values in the implant bodies were recorded in Model 1. In this model, approximately 110.5 MPa of σ_VM_ was measured in the implant located in the premolar region. In Model 2, the maximum stress value observed in the right premolar region implant was approximately 80.0 MPa, which was significantly lower than that in Model 1. In Model 3, the stress levels in the premolar region implants decreased even further, reaching approximately 63.6 MPa. Although the five-implant asymmetric configuration in Model 2 reduced the overall stress levels compared to the four-implant configuration in Model 1, relatively higher stress values were still observed on the right side of the maxilla, where fewer implants were placed, particularly around the implant located in the premolar region.

Model 1 also exhibited significantly higher stress in the abutments, particularly in the posterior region. Notably, in Model 1, which had fewer implants, the stress levels in both the implant bodies and the abutments were significantly higher compared to the other models. As the number of implants increased, stress on the abutments decreased. Stress values in the abutments were similar in Models 2 and 3, although the right-side components in Model 2 consistently showed higher stress than the left.

The maximum stress values in the abutments were generally observed in the implants located in the premolar region across all models and showed similar magnitudes, ranging approximately from 24 to 29 MPa. In contrast, the stress distribution in the framework varied significantly depending on the number of implants. In Model 1, approximately 45.4 MPa was measured in the premolar region, whereas this value was around 18.9 MPa in Model 2 and only 14.2 MPa in Model 3. When the σ_VM_ distribution in the prosthetic framework and crowns was evaluated, the maximum stress values were found to be highest in Model 1. In all models, the stress observed in the prosthetic components tended to decrease toward the posterior region. In the framework, stresses were especially concentrated in the anterior region, particularly on the buccal-facing cervical surfaces. In the crowns, stress accumulation was primarily observed on the palatal cervical surfaces of the anterior region. These findings are presented in Fig. 12.

These results suggest that Model 3 provided the most favorable stress distribution, resulting in the lowest mechanical loads on both the bone and the prosthetic components.

## Discussion

In implant-supported restorations, functional and parafunctional forces generated during mastication are transmitted through the prosthetic superstructure to the implants and peri-implant supporting tissues. These forces may lead to deformation of the surrounding bone and generate stress concentrations at the implant–bone interface, potentially compromising osseointegration and long-term stability [32]. Mechanical stress plays a fundamental role in maintaining bone homeostasis. In animal models, occlusal overload has been identified as a risk factor for peri-implant bone loss [33, 34].

FEA is a widely used method in implant dentistry to assess stress distribution in the peri-implant bone and prosthetic components. Numerous FEA studies have shown that stress distribution is influenced by variables such as implant number, length, and diameter, thread geometry, material properties, and bone quality and quantity [35, 36]. In the present study, FEA was used to compare strain and a stress distributions in cortical and trabecular bone, implants, abutments, and prosthetic structures across three treatment concepts for the atrophic maxilla: All-on-Four, and All-on-Five and All-on-Six approaches incorporating pterygoid implants.

FEA studies commonly utilize stress measures such as σ_VM_, σ_min_ and σ_max_. While σ_VM_ is suitable for ductile materials like metals, principal stress values are more appropriate for brittle structures such as bone [26, 37]. In the present study, since bone exhibits both brittle and ductile properties, principal stresses were used to evaluate bone tissue, whereas σ_VM_ values were applied to assess the prosthetic and implant components.

Bone tissue does not possess homogeneous density and structure, and it is not isotropic [35]. Due to the complex architecture of the jawbones, creating highly accurate geometric models is challenging. Therefore, as in most FEA studies, all materials in the present study were assumed to be homogeneous, isotropic, and linearly elastic [38–41]. A 100% osseointegration rate was also assumed, consistent with previous FEA literature [26, 39].

Studies have shown that differences in stress patterns across models become more evident under oblique loading compared to vertical loading [42]. Moreover, the use of combined loading has been recommended, as it more realistically reflects the direction of masticatory forces and generates higher stress on the cortical bone [38]. Therefore, in this study, oblique forces at a 45° palatobuccal angle were applied bilaterally to simulate masticatory loading on canines, premolars, and molars, consistent with clinical loading directions.

The All-on-Four concept offers an effective rehabilitation approach for completely edentulous jaws with limited bone volume, providing advantages such as shorter treatment duration, lower cost, reduced patient morbidity, and improved quality of life [43, 44]. Although implant survival rates have been reported to exceed 99% in the long term [43–45], prosthesis survival rates tend to decline to approximately 95% at 10 years [46].

Mechanical complications such as prosthetic fractures, porcelain crown damage, and abutment or screw loosening may lead to decreased prosthesis survival, particularly in cases involving excessive loading factors such as bruxism or the presence of long cantilevers. Posterior bone atrophy may necessitate the use of cantilevers, which has been associated with increased mechanical failure rates of up to 50%. Extending posterior support through the placement of additional implants, such as pterygoid implants, can reduce cantilever length and distribute occlusal loads more evenly, thereby improving prosthetic longevity [30, 45]. Biological complications, most commonly the loss of at least one implant, are followed by peri-implant mucositis and peri-implantitis [47]. In the event of implant failure, additional surgical interventions, new implant placement, or augmentation procedures may be required to reconstruct the fixed prosthesis. This not only prolongs the treatment duration but also negatively affects patient compliance and satisfaction, leading to increased costs. Considering all these prosthetic and biological complications, treatment approaches involving a greater number of implants, more balanced load distribution, and enhanced posterior support may be considered more appropriate for improving long-term clinical success. Although previous studies have compared the All-on-Four and All-on-Six concepts [30, 48, 49], no study to date has simultaneously evaluated All-on-Four, All-on-Five, and All-on-Six configurations using pterygoid implants, as conducted in the current investigation.

In Model 1, the highest σ_VM_ values were observed both in the implants and prosthetic components, with stress concentrations particularly noted in the neck regions of the posterior implants and within the prosthetic components. This result is consistent with previous studies indicating that fixed prosthetic systems supported by fewer implants tend to be exposed to higher stress levels. The increased stress is attributed to the transmission of occlusal forces over a smaller contact area, which amplifies the cantilever effect due to limited posterior support [32, 36]. When evaluating the stress distribution in the prosthetic components, including abutments, frameworks, and crowns, the cervical regions of the anterior area appeared as the primary sites of stress accumulation. Model 1 exhibited the highest σ_VM_ values in both the framework and the crowns. As the number of implants increased in Models 2 and 3, the stress values in these components significantly decreased. These findings suggest that implant number directly affects not only the stress distribution in bone tissue but also the load-bearing behavior of the prosthetic superstructure.

In Models 2 and 3, the σ_min_ and σ_max_ in the cortical and trabecular bone were found to be similar; however, stress levels were notably lower in the six-implant configuration. In a FEA comparing four and six-implant models, Bhering et al. reported that increasing the number of implants reduced σ_max_ in the cortical bone [30]. Likewise, Almeida et al., in a study modeling short and tilted implants, found that the All-on-Six concept resulted in lower cortical bone stress [48]. Our findings are consistent with these reports. The greater number of implants in Model 3 may have facilitated a more effective transmission of occlusal forces to the surrounding bone. A similar trend was observed in the trabecular bone, where σ_max_ was lower in both the All-on-Six and All-on-Five groups. This stress reduction with the addition of posterior implants is in line with previously published studies [11, 30, 32]. When used to complement the All-on-4 technique, placing a single pterygoid implant in the posterior region of each maxillary quadrant eliminates the need for distal cantilevers, extends posterior occlusal support, facilitates full-arch rehabilitation, and reduces prosthesis-related complications.

According to physiological thresholds for bone, cortical bone is considered overloaded when σ_min_ exceeds 170 to 190 MPa or σ_max_ exceeds 100 to 130 MPa. In cancellous bone, overloading occurs when either σ_min_ and σ_max_ exceeds 5 MPa [30, 50]. Based on these values, none of the treatment concepts evaluated in this study produced stress levels high enough to be considered pathological for bone tissue.

Although six-implant-supported fixed full-arch restorations have shown predictable long-term outcomes, in some patients with severe bone resorption, the buccopalatal bone dimensions in the anterior maxilla may be insufficient to accommodate even narrow-diameter implants [51, 52]. Furthermore, the remaining bone in this region may not provide the necessary inter-implant distance for placing four implants, or may be unsuitable for placement due to anatomical limitations caused by resorption [53]. Therefore, in this study, a five-implant configuration was evaluated, in which full-arch rehabilitation was achieved using three anterior implants and two pterygoid implants in cases of severely atrophic maxilla. This design was compared with both the All-on-Four concept and the All-on-Six approach incorporating pterygoid implants.

In Models 2 and 3, a reduction in σ_VM_ values was observed with the increased number of implants. In Model 2, the asymmetric distribution with fewer implants on the right side resulted in elevated stress levels in both the cortical bone and the prosthetic components in that region. This finding suggests that asymmetric clinical planning may lead to overloading in the posterior maxilla and cause imbalance in load distribution. These results underscore the importance of symmetrical and well-balanced implant placement to achieve optimal stress distribution. The homogenous stress pattern observed in Model 3 in particular highlights the biomechanical advantage of optimizing implant positioning to support both anterior and posterior regions effectively.

In the present study, maximum principal strain was used to evaluate the biomechanical behavior of peri-implant bone, as it reflects tensile deformation, which plays a critical role in bone remodeling and potential failure. According to the mechanostat theory, strain values between 300 µε and 1000 µε are considered within the physiological loading range that supports bone homeostasis [54]. Across all three models, strain values remained within the physiological range, suggesting that none of the loading conditions produced excessive mechanical stress on the peri-implant bone. This finding indicates that all treatment protocols examined in this study are biomechanically safe with respect to strain-induced bone risk. Among the models, Model 1 demonstrated the highest maximum principal strain values, particularly in the buccal bone surrounding the distal implants. This concentration is likely associated with the cantilever effect due to the limited posterior support in the All-on-Four configuration. In contrast, Model 3 exhibited a more favorable and evenly distributed strain pattern, especially in the premolar and pterygoid implant regions, highlighting the biomechanical advantage of extended posterior support. These results are consistent with previous reports indicating that the addition of pterygoid implants effectively reduces posterior bone stress and strain by facilitating better distribution of occlusal loads [55–57]. Overall, the strain analysis supports the biomechanical validity of all three implant configurations. Nevertheless, configurations incorporating pterygoid implants may offer enhanced mechanical behavior by reducing localized strain concentrations, thereby potentially improving long-term bone preservation and implant success in the atrophic maxilla.

These results are consistent with previous FEA-based investigations that demonstrated similar stress and strain patterns in posterior implant configurations, including those incorporating pterygoid implants, thereby supporting the validity of our model and confirming its clinical relevance [20, 21, 23, 24, 56].

The biomechanical stress and strain distribution patterns observed in this study have important implications for bone remodeling and long-term implant stability. In this study, all models exhibited strain levels within the physiological range (300–1000 µε), which supports bone maintenance and remodeling [54]. However, localized increases in tensile strain, particularly in the posterior region of the four-implant configuration, suggest a potential risk for overload. Such conditions may impair osseointegration and increase the risk of marginal bone loss. In contrast, the six-implant configuration demonstrated a more balanced and favorable strain distribution, supporting biomechanical stability and adaptive bone response. These findings highlight the clinical importance of optimizing implant number, position and enhancing posterior support to reduce biomechanical risk and improve long-term outcomes.

The findings of this study are in line with prior literature, which highlights the mechanical benefits of posterior support in the atrophic maxilla. Wilkirson et al. [23] demonstrated that models incorporating pterygoid implants exhibited lower stress and strain in surrounding bone compared to control models lacking posterior support, emphasizing their biomechanical advantage. Similarly, a recent study by Darwish et al. [56] explored different configurations involving zygomatic and pterygoid implants, revealing that the inclusion of pterygoid implants led to reduced peri-implant bone stress, lower abutment-level stress, and decreased micromovement. These findings confirm that pterygoid implants enhance mechanical stability by improving load distribution. Consistent with these observations, our study found that the addition of pterygoid implants in Models 2 and 3 contributed to more balanced stress and strain distribution, particularly in the posterior regions, compared to the All-on-Four model (Model 1). This suggests that pterygoid support not only reduces cantilever-induced biomechanical risk but may also improve the long-term prognosis of full-arch rehabilitations by maintaining physiological strain levels in peri-implant bone.

The stress and strain patterns observed in this study have important clinical implications regarding the use of pterygoid implants and offer useful guidance for treatment planning in atrophic maxilla cases. The elevated stress and maximum principal strain values found in the four-implant configuration, particularly around the buccal bone of the distal implants, highlight the potential biomechanical disadvantages of long cantilevers. Such configurations may increase the likelihood of prosthetic fractures or peri-implant bone loss in real-world settings due to localized mechanical overload. In contrast, the addition of pterygoid implants in the five- and six-implant models improved posterior load distribution and resulted in more favorable strain patterns, remaining within physiological limits [22, 23]. This suggests improved bone preservation potential and reduced risk of biomechanical failure. Recent findings by Vinodh et al. [24] further support this biomechanical advantage; their comparative analysis of All-on-4 and pterygoid-supported full-arch prostheses using strain gauges and finite element methods revealed more efficient load transfer and lower stress values in the pterygoid implant group. This underscores the clinical utility of posterior support in minimizing cantilever-related complications. Accordingly, incorporating pterygoid implants may represent a practical, graftless alternative to conventional sinus lifting or bone augmentation procedures, offering improved biomechanical conditions without increasing surgical morbidity. These insights reinforce current treatment principles advocating for optimized implant distribution, particularly by emphasizing the clinical relevance of pterygoid implants in modern full-arch treatment concepts for the atrophic maxilla. They may also encourage clinicians to consider graftless posterior anchorage alternatives. Therefore, clinicians may consider optimizing implant number and distribution to preserve marginal bone, enhance implant longevity, reduce biomechanical complications, and support long-term prosthetic success.

The results of this study emphasize that, in implant placement planning, not only the number of implants but also factors such as symmetry, angulation, and posterior support play a critical role. Pterygoid implants contributed significantly to stress reduction and improved balance across all evaluated parameters. From a clinical standpoint, these findings support the use of pterygoid implants as a viable alternative in cases of severe maxillary atrophy.

One of the main limitations of this study is the use of an idealized anatomical model, which may not fully reflect biological variability, differences in bone quality, or complex prosthetic biomechanics. A single, standardized maxillary model was used to allow consistent comparison of implant configurations. Although patient-specific geometries may affect absolute stress values, the relative performance trends among the configurations are expected to remain consistent. Moreover, all materials were assumed to be linearly elastic, homogeneous, and isotropic, which does not fully capture the anisotropic and heterogeneous nature of human bone. This assumption may influence the accuracy of simulated stress and strain distributions. Another important limitation is the absence of experimental or clinical validation, which is a common constraint in FEA-based studies. Moreover, static loading conditions were used, which do not capture dynamic masticatory forces. Finally, since FEA is inherently time-independent, this study cannot predict time-dependent phenomena such as bone remodeling, osseointegration dynamics, or long-term prosthetic wear. Future studies should aim to address these limitations by incorporating patient-specific anatomical data, dynamic loading scenarios, and experimental or clinical validation to enhance the physiological relevance and clinical applicability of the findings.

## Conclusion

In conclusion, this study demonstrated that both implant number and placement strategy are critical determinants of stress distribution in the bone and prosthetic components of the atrophic maxilla. Although all models exhibited stress and strain values within physiological limits, configurations incorporating pterygoid implants showed more favorable biomechanical performance. Increasing the number of implants resulted in a more uniform stress distribution, while symmetrical placement enhanced stability and prosthesis longevity. The addition of pterygoid implants provided improved posterior anchorage, reduced cantilever effects, and facilitated balanced load transfer. These findings provide biomechanical insights that may support the clinical consideration of pterygoid implants in treating the severely atrophic maxilla, especially in cases with limited posterior bone. Overall, the outcomes contribute to the biomechanical understanding necessary for optimizing implant-supported full-arch rehabilitations.

## Data Availability

No datasets were generated or analysed during the current study.

## Abbreviations

FE: Finite element
FEA: Finite element analysis
σ_VM_: von Mises stress
σ_max_: Maximum principal stress
σ_min_: Minimum principal stress
µε: Microstrain
